# Nutritional status of children 7–36 months old from millet consuming communities of Masindi District, Western Uganda

**DOI:** 10.1186/s40795-019-0273-z

**Published:** 2019-02-11

**Authors:** Barugahara Evyline Isingoma, Samuel Kuria Mbugua, Edward Gichohi Karuri

**Affiliations:** 1 Department of Food Science, Nutrition and Technology, University of Nairobi, Nairobi, Kenya; 2 Department of Human Nutrition and Home Economics, Kyambogo University, Kampala, Uganda

**Keywords:** Nutritional status, Millet consuming, 7–36 months, Western Uganda

## Abstract

**Background:**

Several national reports have indicated poor nutritional status among children from Western Uganda where millet porridge is a predominant complementary food. However, little is known about the nutritional status of 7–36 months old children from millet consuming communities of Western Uganda.

**Methods:**

A cross-sectional study was conducted in Bujenje County of Masindi District. A total of 636 children from 23 villages within Bwijanga and Budongo sub counties were randomly selected. Anthropometric measurements of children were taken. Data on demographic and socioeconomic characteristics of children’s households, their dietary practices and morbidity patterns was collected using a self-administered questionnaire. A statistical Package for the Social Sciences (SPSS) version 20 and Emergency Nutritional Assessment (ENA) Software Version 2010 were used for analysing data. The relationship between demographic and socioeconomic characteristics of households and children’s nutritional status was determined using Chi-square tests. Pearson’s correlation coefficient was used to determine the association between children’s nutritional status and the amount of millet porridge consumed. A *p*-value of < 0.05 indicated statistical significance.

**Results:**

A proportion of 30.5% children were stunted, 11.6% underweight and 7.4% wasted. Underweight and wasting were significantly high in Budongo sub county at *p* = 0.044 and *p* = 0.005 respectively. Stunting and underweight were highest between 12 and 23 months at *p* = 0.005 and 0.020 respectively. Although millet porridges formed the bulk of children’s meals, they could only cater for < 60% of the recommended daily nutrient intake. Children with diarrhoea were 1.4 and 2 times likely to become stunted and underweight at *p* = 0.025 and 0.007 respectively. Feeding practices for children with diarrhoea were contrary to World Health Organisation’s recommendations in more than 50% of the studied children. There was a significant association between Height-for-Age Z scores, Weight-for-Height Z scores and the amount of millet porridge consumed by children (*r* = − 0.20, *p* < 0.001 and *r* = 0.14, *p* < 0.001 respectively).

**Conclusions:**

Results showed slightly higher percentages of stunted, underweight and wasted children compared to national figures. This was attributed to high incidences of diarrhoea and inadequate feeding practices especially for children 12–36 months old.

## Introduction

Child malnutrition is a public health problem since it is a major contributing factor to mortality, morbidity, intellectual and physical disability among children [1]. The first 3 years of life are the most critical in the development of children due to the high nutrient requirements, limited gastric capacity and low immunity during this period [2]. The consequences of inadequate nutrient intake during this period may result into irreversible damages to the mental and physical development of children [3]. Globally, an estimated 165 million, 10 million and 52 million of children below 5 years are stunted, underweight and wasted respectively [4]. Africa and Asia account for more than 90% of the world’s stunted children, with Sub-Saharan Africa having a prevalence rate of 36% compared to 56% for Asia [4]. About 29% of children less than 5 years in Uganda are stunted, 11% underweight and 4% wasted [5]. The Uganda Demographic and Health Survey reports have persistently ranked the Western region of Uganda as a very high prevalence area for child stunting with some districts having a percentage prevalence of above 30% [5–7].

Masindi District is one of the 5 districts in Bunyoro sub region found in Western Uganda. Recent Demographic and Health Survey reports indicate 34.5% stunting, 9.1% underweight and 3.8% wasting rates among children below 5 years in Bunyoro sub region. Data on the nutritional status of children below 5 years in Masindi District specifically is scanty. However some studies have indicated that 22% of the children under 5 years were found to be underweight in 2006 [8].

Millet porridge is a common complementary food in Western Uganda [9, 10]. Finger millet *(Eleusine coracana)* is recommended as a weaning food because it is one of the most antiallergenic, easy to digest and nutritious foods among cereals [11]. However, being plant based, it is limited in essential amino acids, has low energy and nutrient density and a high content of ant nutrients such as phytates and tannins [12]. Its prominent use has been attributed to the existence of high levels of child malnutrition among some communities [13]. Ant nutrients in millet reduce the bioavailability of vitamins and minerals and contribute to poor digestibility of starch and protein and this makes young children relying on it vulnerable to malnutrition [12, 14].

A survey carried out in Masindi District found out that inadequate nutrient intake and infections especially diarrhoea and malaria were common among children aged 7–24 months [10]. According to the UNICEF conceptual framework, inadequate dietary intake and diseases in children immediately manifest into poor nutritional status. The current study was aimed at generating information about nutritional status and feeding practices among millet consuming communities. The specific objective of this study was to identify the association between nutritional status and household socio-demographic, morbidity patterns and feeding practices among children 7–36 months old from millet consuming communities in Masindi District.

## Methods

### Study setting

The study was cross-sectional and analytical. Masindi District was purposively chosen because 53.1% of children aged 7–24 months consume millet porridge [10], it is also one of the most rural based districts in Western Uganda and it is characterised by low income households and common occurrence of infections among children [8]. Two highest millet producing sub counties in Masindi District namely Budongo and Bwijanga were chosen [8]. Fisher’s Equation [15] was used to calculate the sample size: n = Z^2^pq/d^2^. Where n = minimum sample size, z = the standard normal deviate at 1.96 for a confidence level of 95%, p = proportion of stunted children below 5 years in Western Uganda [5], q = the proportion of children below 5 years who are not stunted in Western Uganda, d = the degree of accuracy desired, set at 0.05. The study subjects and respondents were children aged 7–36 months and their mothers/caretakers respectively. A sample size of 636 households was recruited by random cluster sampling method. Children aged 7–36 months were recruited from 23 villages (*n* = 10 in Budongo and *n* = 13 in Bwijanga). This matched the national distribution and was representative of Bujenje County of Western Uganda [8]. Only the youngest child in the household was included in the study and in case of twins in the household, both were included for cultural reasons. Each household was visited twice over a period of 1 month and only children from households with mothers/caretakers present were recruited.

### Data collection and management

Self-administered questionnaires were used to collect data on socioeconomic and demographic characteristics of households as well as morbidity status and dietary practices of children.

Anthropometric measurements were taken using calibrated equipment and standardized techniques [16]. Standing height/ length was taken using Short’s Height Measuring Board (Short Productions, Woonsocket, RI) and recorded to the nearest 0.1 cm. Body weight was taken using light weight SECA mother–infant scales with a digital screen that were designed and manufactured under the guidance of UNICEF. Mid Upper Arm Circumference (MUAC) was measured basing on WHO guidelines and recorded to the nearest 0.1 cm [17]. All measurements were taken in duplicate with the children in light clothing and without shoes. Each measurement was taken by the same person to eliminate inter examiner error.

### Statistical analysis

Data was entered, cleaned and analyzed using SPSS (Statistical Package for Social Scientists) version 20.0 for windows. Nutritional status indices for Height-for-Age (HAZ), Weight-for-Age (WAZ), Weight-for-Height (WHZ) and Mid Upper Arm Circumference (MUAC) were calculated using ENA for SMART 2010 software and interpreted using WHO’s 2006 reference standards. The nutrient intake derived from the porridges was determined basing on laboratory results from our previous study [18] and compared with World Health Organisation’s (WHO) recommendations [19]. The Body Mass Index (BMI) of mothers was categorised basing on WHO recommendations [6] as follows;

**Table:** 

Value	Classification
< 18.5	Underweight
18.5–24.9.	Normal
25–29.9	Overweight
30 and above	Obesity

Descriptive statistics were compiled. Pearson’s Chi-square tests using Cross tabulations were done to establish the influence of demographic and socioeconomic characteristics, and morbidity patterns on children’s nutritional status. Correlation between nutritional status and amounts of millet porridges consumed by children was established using Pearson’s correlation coefficient analysis. A *p*-value of < 0.05 was accepted as indicating statistical significance.

### Ethical approval

This study was reviewed by The Aids Support Organisation (TASO) internal review board (TASOIRC/029/13-UG-IRC-009) and approved by the Uganda National Council of Science and Technology (HS 1315). Verbal and written consent were obtained from the parents of the study children.

## Results

### Socioeconomic and demographic characteristics of the population studied

Data was collected from 636 children and their mothers/caretakers. The biggest population of sampled children (58.6%) were from Bwijanga Sub County (Table 1). Nearly half (46.4%) of all the households had a total household income ranging from $10–14.9 per year. We could not collect data on household income for 58 households because they were not willing to disclose their income. More than half of the children (54.4%) were males and 41.7% of the children studied were aged 12–23 months. Slightly more than half (57.9%) of the children were either first or second born. We were not able to establish the birth order of two of the study children because their caretakers lacked this information. Most of the children studied (91.8%) were taken care of by their mothers. Almost 81% of the mothers were married and 72.4% had completed primary education.

**Table 1:** Demographic and socioeconomic characteristics of the population studied

Parameter	Number	Percent
Sub County		
Bwijanga	263	58.6
Budongo	373	41.4
Household income per year ($)		
10–14.9	295	46.4
15–19.9	178	28.0
20–24.9	80	12.6
25–30	25	13.0
Sex of studied children		
Male	346	54.4
Female	290	45.6
Age of children (months)		
7–8	60	9.4
9–11	65	10.2
12–23	265	41.7
24–36	246	38.7
Birth order		
1–2	367	57.9
3–4	162	25.6
> 4	105	16.5
Caretaker of the index child		
Mother	584	91.8
Grandmother	31	4.9
Aunt	21	3.3
Mother’s marital status		
Not married	109	18.7
Married	475	81.3
Mother’s education level		
Informal education	53	8.3
Primary	460	72.4
Secondary	123	19.3
Mothers Body Mass Index (BMI)		
< 18.5 (Underweight)	163	27.9
18.5–24.9 (Normal)	370	63.4
25–29 (Overweight)	51	8.7

### Nutritional status of the studied children

Table 2 shows the nutritional status of children in Bujenje County. Stunting, underweight, wasting and under nutrition by MUAC among children in the study area were 30.5, 11.6, 7.4 and 6.9% respectively. Stunting was higher in Bwijanga Sub County (33%) compared with Budongo Sub County (27.1%) but the difference was not significant. Underweight was significantly higher in Budongo Sub County (14.8%) than Bwijanga Sub County (9.4%) at *p* = 0.044. Similarly, wasting was significantly higher in Budongo Sub County (11%) than Bwijanga Sub County (4.8%) at *p* = 0.005. Under nutrition as determined by MUAC, was also significantly higher in Budongo Sub County (9.1%) than Bwijanga Sub County (5.4%) at *p* = 0.030. Stunting and underweight significantly increased after 12 months but reduced later at 24–36 months (*p* = 0.005 and 0.020 respectively). Males were significantly more stunted and underweight than females at *p* < 0.001. The percentage of malnourished children (stunting, underweight and wasting) reduced with increasing household size. The significant reduction was observed in underweight at *p* = 0.042 (Table 3).

**Table 2:** Prevalence of stunting, underweight and wasting in Bujenje County

Nutritional status	Sub Counties		Bujenje County n (%)	*p*-value
	Budongo n (%)	Bwijanga n (%)		
Stunting (<−2 z-scores)	71 (27.1)	123 (33.0)	194 (30.5)	0.063
Moderate > − 3 to < − 2 z-scores	55 (20.9)	91 (24.4)	146 (23.0)	
Severe <−3 z-scores	16 (6.1)	32 (8.6)	48 (7.5)	
Underweight (<−2 z-scores)	39 (14.8)	35 (9.4)	74 (11.6)	0.044
Moderate > − 3 to < −2 z-scores	30 (11.4)	31 (8.3)	61 (9.6)	
Severe <−3 z-scores	9 (3.4)	4 (1.1)	13 (2.0)	
Wasting (<−2 z-scores)	29 (11)	18 (4.8)	47 (7.4)	0.005
Moderate (> − 3 to < −2 z-scores)	24 (9.1)	14 (3.8)	38 (6.0)	
Severe (<−3 z-scores)	5 (1.9)	4 (1.1)	9 (1.4)	
MUAC				
Severe (< 11.5)	50 (1.9)	4 (1.1)	9 (1.4)	
Moderate (11.5–12.4)	19 (7.2)	16 (4.3)	35 (5.5)	0.030
At risk (12.5–13.5)	29 (11.0)	23 (6.2)	52 (8.2)

**Table 3:** Nutritional status according to child characteristics and household size

Variable	Stunting		Underweight		Wasting	
	% prevalence	Mean HAZ	% prevalence	Mean WAZ	% prevalence	Mean WHZ
Age (months)						
7–8	2.5	2.67 ± 0.78	2.0	2.75 ± 0.07	0.9	2.90 ± 0.05
9–11	1.2	2.85 ± 0.05	0.9	2.89 ± 0.04	0.6	2.94 ± 0.03
12–23	14.5	2.56 ± 0.04	5.5	2.84 ± 0.03	3.9	2.89 ± 0.02
24–36	12.3	2.61 ± 0.04	3.2	2.89 ± 0.03	2.0	2.93 ± 0.02
*p*-value	0.005	0.009	0.020	0.059	0.329	0.490
Sex						
Male	10.5	2.54 ± 0.04	3.1	2.81 ± 0.03	4.3	2.91 ± 0.02
Female	20.0	2.71 ± 0.043	8.5	2.92 ± 0.02	3.1	2.91 ± 0.02
*p*-value	0.001	0.017	0.001	0.001	0.760	0.910
Household size						
< 5	16.5	2.56 ± 0.04	6.8	2.83 ± 0.03	4.7	2.89 ± 0.02
5–7	9.4	2.70 ± 0.04	2.8	2.91 ± 0.02	1.9	2.94 ± 0.02
Above 7	4.6	2.62 ± 0.06	2.0	2.85 ± 0.04	0.8	2.93 ± 0.03
*p*-value	0.084	0.040	0.042	0.020	0.089	0.170

Table 4 shows that majority of the stunted and underweight children had diarrhea 2 weeks prior to the study and children with diarrhea were 1.4 and 2.0 times likely to become stunted and underweight at *p* = 0.025 and *p* = 0.007 respectively.

**Table 4:** Nutritional status basing on prevalence of diarrhea in the previous 2 weeks

Nutritional status	Diarrhea in the last 2 weeks		OR (95% CI)
	Yes (n) %	No (n) %	
Stunted	124 (63.9)	70 (36.1)	1.437 (1.015–2.035),
Normal	244 (55.2)	198 (44.8)	*p* = 0.025
Underweight	53 (71.6)	21 (28.4)	1.979 (1.162–3.369),
Normal	315 (56.0)	247 (44.0)	*p* = 0.007
Wasted	25 (53.2)	22 (46.8)	0.815 (0.449–1.479),
Normal	343 (58.2)	246 (41.8)	*p* = 0.300

### Nutrient intake from millet porridges

The mean frequency of feeding children on millet porridges varied from 1.91 ± 0.91 to 3.02 ± 0.05 meals per day. The amount of porridges consumed per day varied from 412.65 ± 3.67 to 906 ± 9.80 ml. The porridges were deficient of vitamin A and they covered less than 60% of the recommended energy, protein, iron and zinc intake (Table 5). There was a significant association between the amount of millet porridge consumed by children per day and children’s Height-for-Age, Weight-for-Height Z scores (*r* = − 0.20, *p* < 0.001 and *r* = 0.14, *p* < 0.001) respectively.

**Table 5:** Nutrient intake from millet porridges

Variable	7–8 months	9–11 months	12–23 months	24–36 months
Frequency of feeding	1.91 ± 0.19	2.13 ± 0.15	2.80 ± 0.07	3.02 ± 0.05
Amount of porridges/day (millilitres)	412.65 ± 3.67	558.12 ± 13.69	841.01 ± 7.94	906.32 ± 9.80
^a^Energy				
Recommended intake	269 k cal	451 k cal	746 k cal	1150 k cal
Total intake	69.5 k cal	156.1 k cal	236.6 k cal	196.4 k cal
% RNI covered	26	35	32	17
Protein intake				
Recommended intake	9.1 g	9.6 g	10.9 g	13 g
Total intake	1.6 g	3.7 g	5.6 g	4.7 g
% RNI covered	18	39	51	36
Vitamin A intake (RE)				
Recommended intake	250 μg	300 μg	300 μg	300 μg
Total intake	0 μg	0 μg	0 μg	0 μg
% RNI	0	0	0	0
Iron intake				
Recommended intake	11 mg	11 mg	11 mg	11 mg
Total intake	1.8 mg	4.0 mg	6.0 mg	5.0 mg
% RNI	16	36	55	45
Zinc intake				
Recommended intake	2.8 mg	2.8 mg	2.8 mg	3 mg
Total intake	0.4 mg	0.9 mg	1.3 mg	1.1 mg
% RNI	14	32	46	37

^a^Energy required from complementary foods assuming average amount of breast milk. Iron: Assuming medium iron bioavailability (10%). Zinc: Assuming moderate bioavailability (30%). References for Recommended Nutrient Intake (RNI): World Health Organisation Recommendation [18]

### Knowledge and healthcare practices for children with diarrhea

More than half of the mothers/caretakers (74.2%) had knowledge about oral rehydration salts and 28.5% of these reported giving Oral Rehydration Salts to their children. Only 69% of the children with diarrhea visited a health facility for treatment. Recommended amounts of food and fluids for children with diarrhea were received by 15.2 and 47.5% respectively as shown in Table 6.

**Table 6:** Knowledge and healthcare practices for children with diarrhoea

Variable	*N* = 368	% = 100
Knowledge of Oral Rehydration Salts (ORS)		
No	95	25.8
Yes	273	74.2
Used ORS for the child with diarrhoea		
Yes	263	28.5
No	105	71.5
Source of treatment for diarrhoea		
Visited a health facility	254	69.0
Used herbs	8	2.2
Self medication	103	28.0
Faith healing	3	0.8
Amount of food offered to a child with diarrhoea		
Same as usual	267	72.6
More than usual (Recommended)	56	15.2
Less than usual	45	12.2
Amount of fluids offered to a child with diarrhoea		
Same as usual	192	52.2
More than usual (Recommended)	175	47.5
Less than usual	1	0.3

## Discussion

### Demographic and socioeconomic characteristics of the studied population

Majority of households in Bujenje County lived on less than $2 per day. This reflects an extreme state of poverty which could negatively influence the nutrition status of children. Child malnutrition in Uganda, just like in most developing countries is strongly associated with poverty [2, 5, 20, 21]. Many mothers were taking care of their own children instead of employing caretakers possibly because this is a rural based community. Such a good practice could enhance proper care which is a key aspect in child growth and development [22]. Reported low levels of education among majority of mothers limit utilisation of the already limited resources to improve children’s nutritional status especially given the fact that more than half of the children were either first or second born, probably mothers lacked experience in childcare [22]. Majority of the mothers of the studied children had a normal Body Mass Index (BMI). This is very crucial if mothers are to care for the health and nutritional needs of their children well. Breast milk production has been reported to be low among malnourished mothers and association of BMI with poor child nutrition status has also been reported [22].

### Prevalence of stunting, underweight and wasting among the studied children

Stunting estimates were higher than the global percentages of 25% while underweight and wasting estimates were less than the global estimates of 15 and 8% respectively [23]. Stunting, underweight and wasting levels were comparable to national figures of 29, 11 and 4% respectively and those of Bunyoro sub region (34.5, 9.1 and 3.8% respectively) of children below 5 years. The levels were also close to 26.6, 13.9 and 10.1% stunting, underweight and wasting respectively reported among children below 5 years in cassava consuming communities of Nambale, Busia of Western Kenya [13]. According to the World Health Organisation classification of children’s nutritional status, such percentages of stunted and underweight children reflect serious malnutrition conditions [3]. The prevalence of wasting among these children is ranked as that which demands for interventions [17]. Stunting represents failure of children to receive adequate nutrition over a long period of time and is affected by recurrent and chronic illnesses [3]. Underweight takes into account both chronic and acute under nutrition and it is an overall indicator of a population’s nutritional health [6]. Wasting represents failure of children to receive adequate nutrition in the period immediately preceding the study and this was due to inadequate food intake or a recent episode of diarrhoea or both. Acutely malnourished children who are not immediately treated are likely to develop clinical conditions requiring hospitalisation. Such conditions can cause irreversible damage to mental development [3]. Many health centres tend to be crowded with severely malnourished children and this presents a heavy cost to governments and individuals in the process of treating complications associated with severe acute malnutrition [3].

Stunting and wasting levels were significantly higher in males than females just like in the national reports [6]. This has been reported by other studies and is quite common in the absence of discriminatory measures, but its mechanism is not clear [24, 25]. However it has also been observed that most girl children are trained to be close to their mothers’ right from the early stages of life for cultural reasons, this closeness could attract some privileges like food, especially when there is shortage of food.

The proportion of stunted and underweight children was highest between the ages of 12–23 months which was the weaning period for most of the studied children. This has been reported to be the common age for stunting in most communities with poor quality complementary foods [2]. Weaning children with less nutritious foods affects them since this age group is characterised by high nutrient requirements and limited gastric capacity [26]. The situation is even made worse when infections set in [10]. Increase of stunting with age is consistent with other studies [27] and might also be due to reduced care as some mothers tend to become very busy with economic activities outside the household as children are weaned [28]. The percentage of children who met the recommended nutrient intake (RNI) tended to decrease with age due to increased nutrient requirements as children grew. Most mothers were still relying heavily on porridges without introducing other foods to children and this could have negatively affected the children.

### Factors influencing the nutritional status of the studied children

Our previous findings among children aged 7–24 months in this area showed that majority of these children had inadequate nutrient intake [10]. Children were fed on bulky plant foods with minimal animal products, fruits and vegetables, contrary to World Health Organisation’s recommendations for complementary feeding [10]. Millet porridges formed the bulk of these children’s feeding and were very thin contributing less than 60% of the daily recommended nutrient intake for energy, protein, iron and zinc. The fact that millet porridges had no vitamin A calls for interventions to provide vitamin A in supplements form in order for children to grow as required. Weight-for Height Z scores (WHZ) were positively associated with the amounts of millet porridges taken by children meaning that these porridges are still key players in improving the nutritional status of children in this community. Millet porridges have been reported by some scholars as the most nutritious food among cereals [11]. However there is need to improve the nutritive value of millet porridges if under nutrition among children living in these places is to be addressed. Height-for-Age Z scores were negatively associated with the amount of porridges taken. This was because majority of stunted children were 12–36 months old, where in addition to porridges, mothers were supposed to give other nutritious solid foods. Majority of stunted and underweight children had diarrhoea in the 2 weeks preceding the study and they were more likely to be stunted with low weight. Although WHO recommends increased intake of both food and fluids when children have diarrhoea, many mothers did the contrary perhaps because of lack of knowledge. Uganda Demographic and Health Surveys have also reported feeding practices for children with diarrhoea as being contrary to World Health Organisation’s recommendations. Infections affect dietary intake and utilization and this has a negative effect on the nutritional status of children [22]. After an acute infection, weight gain may be relatively rapid but linear growth is slower, and where infections occur frequently, linear growth recovery may never occur resulting in persistent stunting [3]. In Uganda diarrhoea is a common cause of under nutrition among children and accounts for about 40–50% lower intake for energy and protein among children [3]. We have previously reported that most mothers kept millet porridges for long periods under unhygienic conditions before feeding the children, such a practice could easily attract infections [10]. Many mothers were also involved in digging or providing casual labour to sugar cane plantations where they carry their children along. Such practices could easily result in children picking infections as their immunity is not yet fully developed [3].

Households with few members had majority of their children underweight especially those households comprising mainly of children. This could be due to the dependency burden since majority of the study population were peasant farmers. It also reflects the aspect of proper childcare which is very important in the early stages of growth and development. A study on the nutritional status of children in Gabon also showed better Weight-for-Height Z scores among children from households with many members [29]. Since young children have limited gastric capacity, there is need for feeding them frequently and this can only be possible with availability of parent/caretaker. Essential practices like being hygienic, visiting a health facility/provider when sick, giving vitamin A supplements as recommended and de-worming can only be possible if caretakers are available**.**

Majority of mothers though knowledgeable about Oral Rehydration Salts (ORS) had poor feeding practices during diarrhoea. This could have been responsible for the high wasting levels compared to national figures especially since the period data was collected was a rainy season when infections are high. Caretakers need to be educated on the recommended healthcare practices during diarrhoea. There is need to get an affordable and sustainable solution to the poor healthcare and feeding practices in these rural communities if good nutritional status is to be achieved among children. This perhaps can be done by improving the value of millet porridge, since it is locally available and affordable by most households.

## Conclusions

The stunting, underweight and wasting levels among children from millet consuming communities in Masindi District are slightly higher than the national figures. This was attributed to high incidences of diarrhoea and inadequate nutrient intake from millet porridges especially for children aged 12–36 months. Association of millet porridges with improved Weight-for-Height Z scores in children implies that they have the potential of improving the nutritional status of children if their nutritional value is improved. However, there is also need for timely introduction of solid foods and addressing aspects of poor healthcare practices.

## Abbreviations

BMI: Body Mass Index
CI: Confidence intervention
ENA: Emergency nutrition assessment
HAZ: Height-for-Age Z score
OR: Odds ratio
RNI: Recommended nutrient intake
SD: Standard deviation
SPSS: Statistical Package for Social Scientists
TASO: The Aids Support Organisation
UNICEF: United Nations’ Children Fund
WAZ: Weight-for-Age Z score
WHO: World Health Organisation
WHZ: Weight-for-Height Z score

## Authors’ information

Not available
